# The Fluoro-Thiazolylhydrazone Compound TSC-3C Inhibits Triple Negative Breast Cancer (TNBC) Cell Line Activity by Promoting Apoptosis, Regulating the MAPK Pathway and Inducing Mitochondrial Dysfunction

**DOI:** 10.3390/ijms21031038

**Published:** 2020-02-04

**Authors:** Jiajia Zhang, Jiajia Dai, Qingxuan Zheng, Shuju Guo, Yanyan Yu, Wenpeng Hu, Yanan Gao, Dayong Shi

**Affiliations:** 1 Key Laboratory of Experimental Marine Biology, Institute of Oceanology, Chinese Academy of Sciences, Qingdao 266071, China; zhangjiajia17@mails.ucas.ac.cn (J.Z.); guoshuju@qdio.ac.cn (S.G.); 2 Laboratory for Marine Drugs and Bioproducts of Qingdao National Laboratory for Marine Science and Technology, Qingdao 266071, China; 3 College of Earth and Planetary Sciences, University of Chinese Academy of Sciences, Beijing 10049, China; 4 State Key Laboratory of Microbial Technology, Institute of Microbial Technology, Shandong University, Jinan 250014, China; daijiajia@sdu.edu.cn (J.D.); qingxuan930628@163.com (Q.Z.); yuyanyan@sdu.edu.cn (Y.Y.); wenpeng19961202@163.com (W.H.); gg37595252@163.com (Y.G.)

**Keywords:** triple negative breast cancer, MDA-MB-231 cells, fluoro-thiazolylhydrazone, MAPK pathway, phosphorylated p44/42 MAPK (ERK1/2), phosphorylated c-Jun N-terminal kinase (p-JNK), apoptosis

## Abstract

Triple negative breast cancer (TNBC) is the most aggressive cancer in women, and despite improved treatments, it remains a major cause of morbidity and mortality. We and others have demonstrated that different hybrid compounds targeting PARP/MAPK or other pathways to inhibit cancer progression may lead to promising therapeutic results. We introduced fluorine to alter the physical properties of the compounds. TSC-3C was one of the generated compounds. Upon treatment with TSC-3C, MDA-MB-231 cell proliferation, invasion, and migration were inhibited. **TSC-3C** induced MDA-MB-231 cell mitochondrial dysfunction and apoptosis, which may be caused by reducing the level of phosphorylated p44/42 MAPK (ERK1/2) and increasing the level of p-JNK. The present study may help to elucidate the role of the MAPK pathway in the development of breast cancer and may promote further research on halogenated heterocyclic compounds for the treatment of breast cancer.

## 1. Introduction

Breast cancer is one of the leading causes of cancer-related mortality in women worldwide, and the rapid increase in the prevalence and mortality rates associated with this disease are worrying [1,2]. Triple negative breast cancer (TNBC) is the most aggressive type of breast cancer. This molecular subtype of breast cancer is characterized by a gene expression profile similar to that of the basal–myoepithelial layer of the normal breast [3]. TNBC is identified as a cancer that lacks expression of the estrogen receptor (ER), progesterone receptor (PR), and HER2. MDA-MB-231 is a triple negative breast cancer (TNBC) cell line [4,5]. TNBC was previously associated with a poor perceived prognosis [6,7]; however, the use of adjuvant capecitabine in combination with docetaxel and cyclophosphamide, plus epirubicin, to treat TNBC patients has led to an increased five-year event-free survival ratio of 86.3% [8]. Current anticancer therapies combine agents with different molecular mechanisms, such as specific drugs and chemotherapies or radiotherapies [9]. Many PARP/MAPK-targeted treatments and other kinds of drugs have been found to be effective, and the effect of MAPK in TNBC has been characterized [10,11,12,13,14,15,16,17,18]. However, the emergence of drug resistance remains a major challenge. There is an urgent need to develop efficient promising agents for TNBC.

Halogenated compounds come from various sources, such as natural marine products [19], and have diverse potent activities, including antidiabetic [20], antimicrobial [21], antioxidative [22], anti-inflammatory [23], and anticancer activities [23,24,25,26,27]. In our previous work, a series of bromophenol hybrids with active anticancer moieties were designed and synthesized as potential anticancer agents [28,29,30]. The chloro-thiazolylhydrazone compound 4EGI-1 is an inhibitor that has been reported to have a structure–activity relationship (SAR) and anticancer activity [31,32]. Thus, we designed two series of thiazolylhydrazone derivatives containing the active moiety of the dimethoxyphenol ring, based on the structure–activity relationships (SARs) identified in our previous research studies [30,31,33]. We introduced fluorine to alter the physical properties and binding characteristics of the compound, to develop novel drugs [34]. While the active group of thiazolylhydrazone was preserved, several fluorine-containing target compounds were designed with the expectation to obtain effective compounds [35].

MAPKs are enzymes that regulate targets within cells by binding to cell-surface receptors, which are evolutionarily conserved. Three-tiered cascades, composed of MAPK, MAPK kinase (MAPKK, MKK, or MEK), and MAPKK kinase or MEK kinase (MAPKKK or MEKK), regulate MAPK activity [36]. Almost all cellular processes, from gene expression to cell death, are regulated by MAPKs. Mammals express at least four distinctly regulated groups of MAPKs, which are extracellular signal-related kinases (ERK)-1/2, Jun amino-terminal kinases (JNK1/2/3), p38 proteins (p38a/b/g/d), and ERK5, which are activated by different MAPKKs [37,38]. Many agents, such as oxidative stress, UV radiation, transforming growth factor-β treatment, and anticancer drugs, induce apoptosis by activating JNK and p38MAPK cascades [39,40]. Other agents suppress apoptosis by inhibiting JNK and p38MAPK. In contrast, ERK is generally considered a survival factor. The Ras-MAPK signaling pathway, by inhibiting the expression and activity of the proapoptotic protein Hid, promotes cell survival [41]. Apoptosis is regulated by a balance between the activation of JNK, p38MAPK, and ERK [42]. Our work on the effect of the balance between JNK and ERK on apoptosis verified the mechanism of action of compound **TSC-3C**.

In this study, we found that MDA-MB-231 cells are sensitive to the thiazolylhydrazone hybrid **TSC-3C**. We investigated it’s in vitro anticancer activity against ten human cancer cell lines and one normal cell line. The lowest half maximal inhibitory concentration (IC_50_) of **TSC-3C** was observed in MDA-MB-231 cells, which make up a typical cell line of TNBC. We aimed to evaluate the anticancer activity of **TSC-3C** and investigate its mechanism. **TSC-3C** inhibited MDA-MB-231 cell proliferation, migration, and invasion in a concentration-dependent manner. The **TSC-3C** hybrids promoted the apoptotic rate by the mitochondria apoptosis pathway. **TSC-3C** may regulate the MAPK pathway to inhibit MDA-MB-231 cells by reducing the phosphorylated extracellular signal-regulated kinase ERK1/2 (phosphorylated p44/42 MAPK) and phosphorylated c-Jun N-terminal kinase (p-JNK). 

Although few potent compounds have been previously reported [28,29,30,35,43], the anti-TNBC activity of fluoro-thiazolylhydrazone hybrids is reported for the first time in this work. The anticancer activity of **TSC-3C** for TNBC is evidence for a promising therapeutic agent that is important for drug discovery and clinical applications.

## 2. Results

### 2.1. Chemistry

**Scheme 1:**
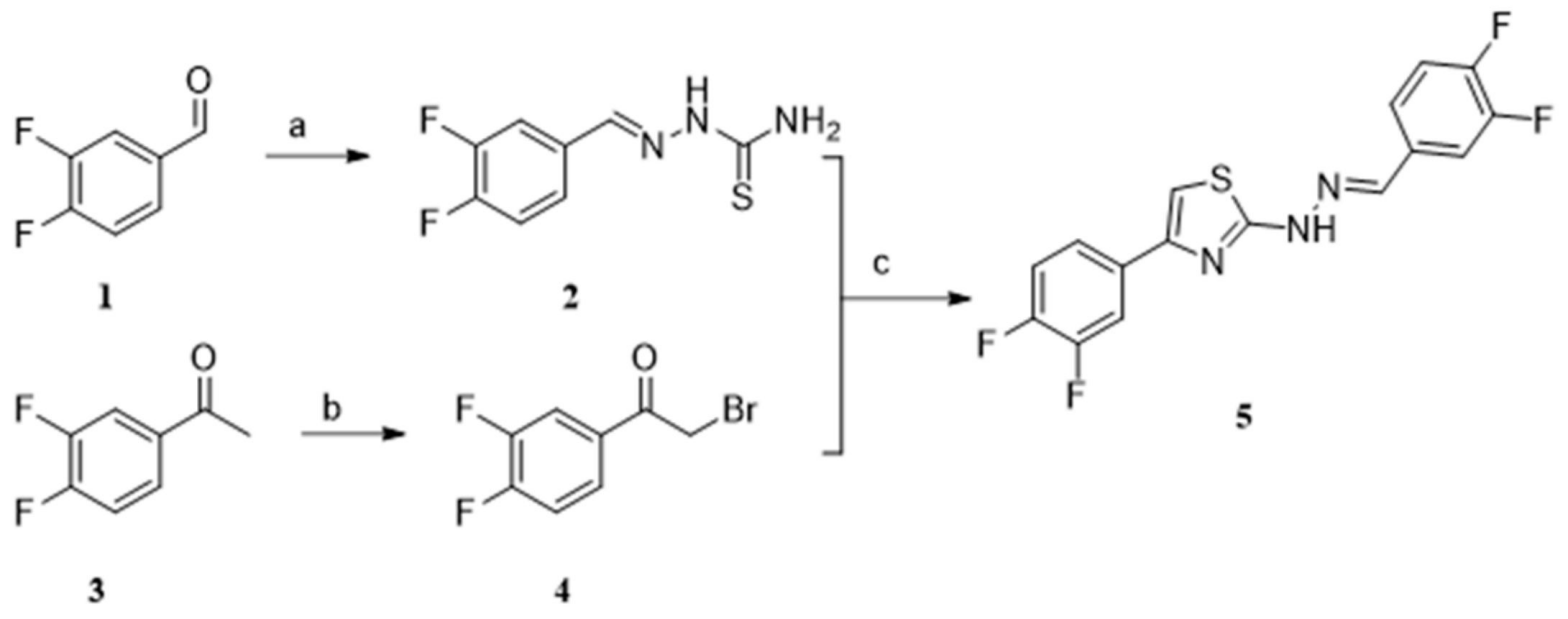
Reagents and conditions: (a) CH3COOH, 95% EtOH, 90 °C; (b) CuBr2, EtOAc, 90 °C; (c) CH3COONa, EtOH, 90 °C.

For the synthesis of (E)-2-(2-(3,4-difluorobenzylidene) hydrazinyl)-4-(3,4-difluorophenyl) thiazole compounds, we prepared (E)-2-(3,4-difluorobenzylidene) hydrazine-1-carbothioamide, compound **2**, and 2-bromo-1- (3,4-difluorophenyl)ethan-1-one, compound **4**, as intermediates (Scheme 1). To this end, a generally established method is the condensation of 3,4-difluorobenzaldehyde, compound **1**, and thiosemicarbazide in ethanol (EtOH, 95%) [44]. Bromination of 3,4-difluoroacetophenone was carried out in ethyl acetate, with copper bromide as the bromination reagent [45]. Target compound **5**, named **TSC-3C**, was synthesized starting from **4** and **2**, under the conditions of ethanol and anhydrous sodium acetate [35].

### 2.2. Anti-Proliferative Activity

The cytotoxicity assessment was performed against eight human cancer cell lines and one normal cell line as follows: A549 (human lung cancer cell line), Caco-2 (human colonic epithelial cell line), HepG2 (human hepatocellular carcinoma cell line), U87 MG (human glioma cell line), MDA-MB-231 (human breast cancer cell line), Capanc-1 (human pancreatic cancer cell line), SK-OV-3 (human ovarian cancer cell line), MCF-7 (human breast cancer cell line), and L-02 (human liver cell line). The experiments were performed by using the 3-(4,5)-dimethylthiahiazo (-z-y1)-3,5-di- phenytetrazoliumromide method (MTT assay). The IC_50_ values of **TSC-3C** in a variety of cell lines are listed in Table 1. 

**Figure 1:**
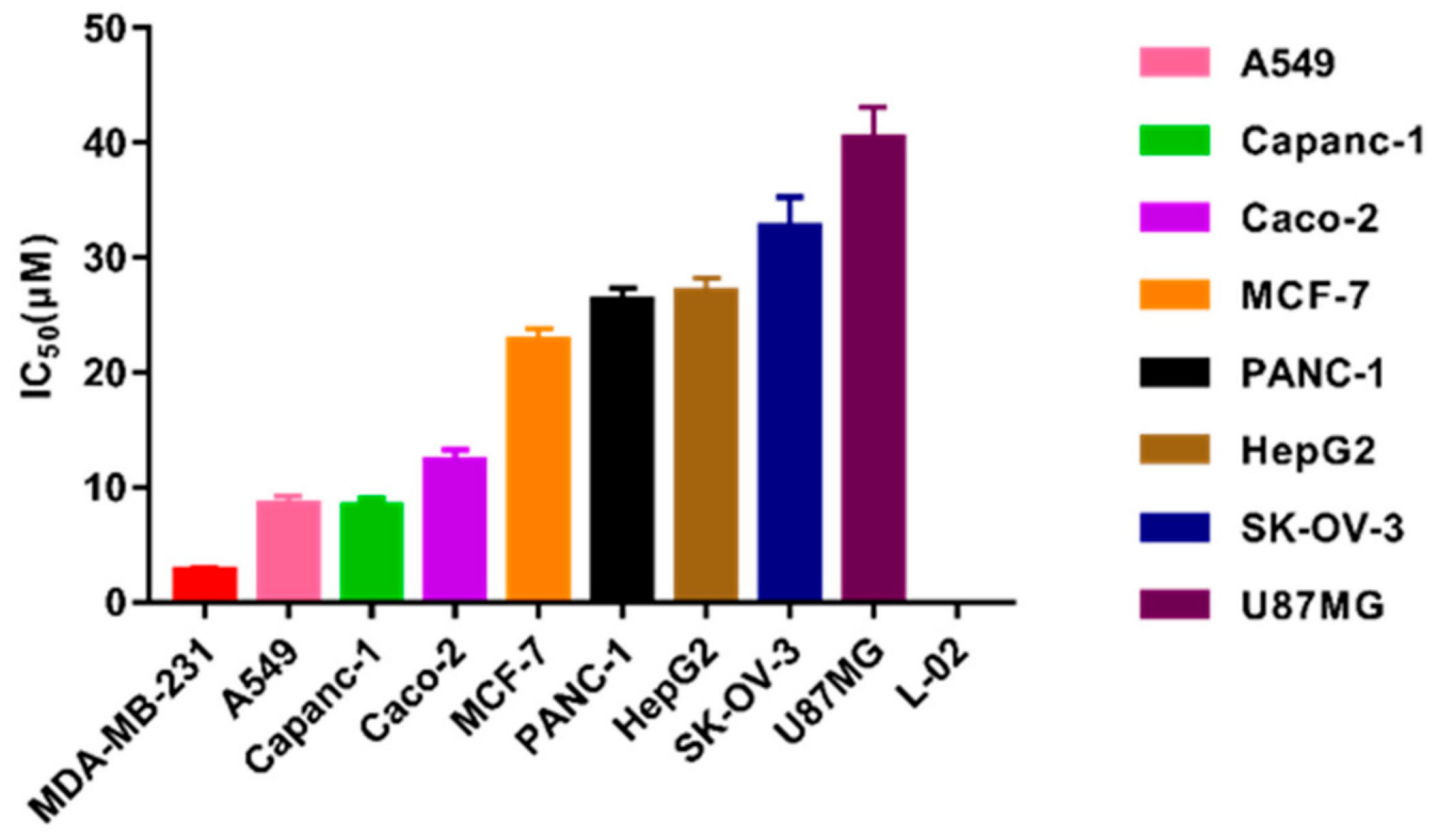
Bar graph of the IC_50_ values in a variety of cell lines.

**TSC-3C** had different inhibitory activities on the proliferation of the tested cell lines, which were dose-dependent. **TSC-3C** significantly inhibited MDA-MB-231 cells more than it did with any of the other cells. The IC_50_ was 2.79 ± 0.71 μM. **TSC-3C** showed no toxicity against L-02, which showed in the Figure 1. The in vitro results of the inhibitory effect on proliferation indicated that **TSC-3C** was a promising agent for breast cancer treatment.

### 2.3. TSC-3C Inhibited the Proliferation of MDA-MB-231 Cells

**Figure 2:**
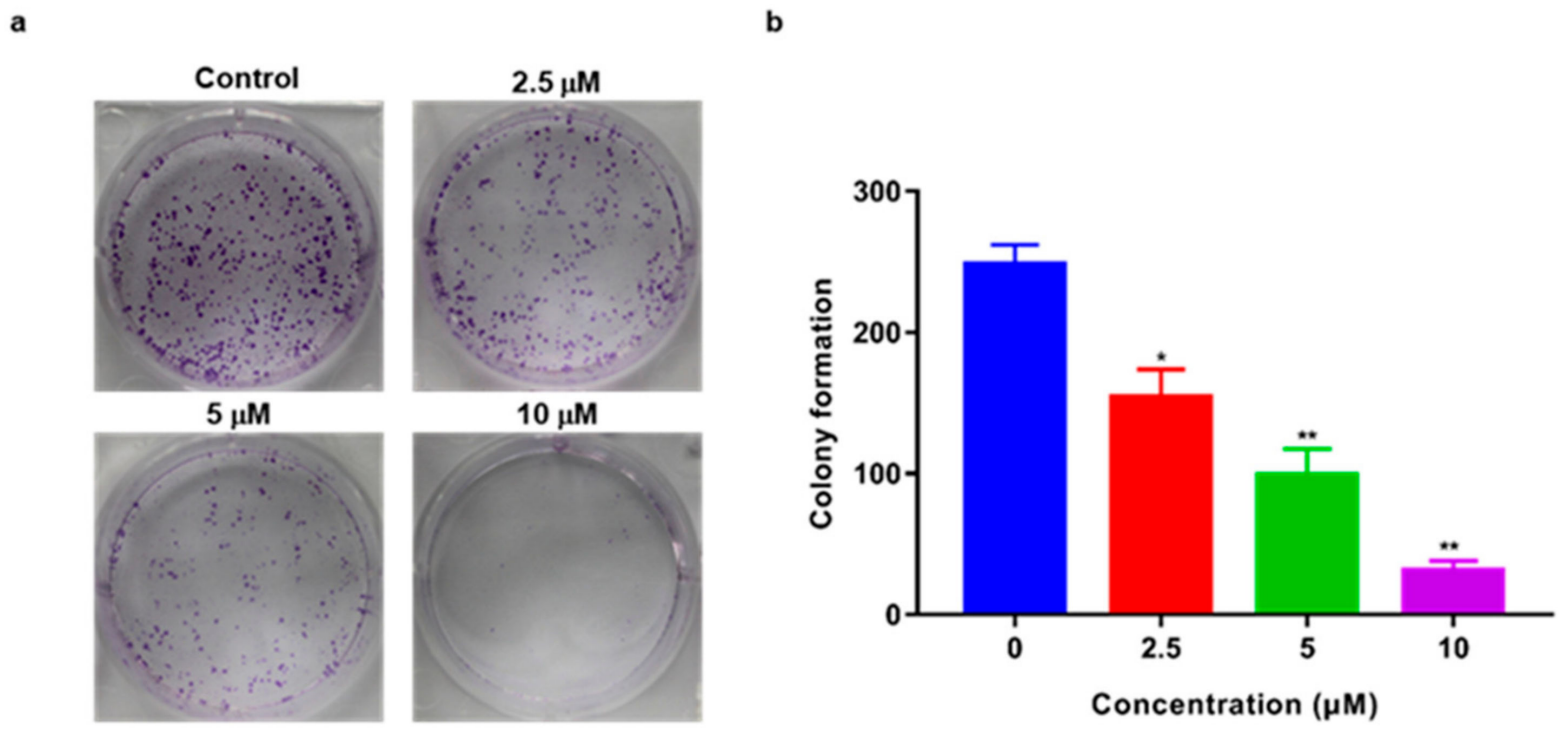
(**a**) Colony formation results of MDA-MB-231 cells cultured with **TSC-3C**; (**b**) Bar graph of colony formation; The data are expressed as the means ± SD from three independent experiments. * *p* < 0.05 vs. the control group, ** *p* < 0.01 vs. the control group.

A population of cells originating from a single cell is called a colony. MDA-MB-231 cells were treated with gradient concentrations of **TSC-3C** for 10 days, and colony formation was stained with crystal violet. As shown in Figure 2, at a concentration of 2.5 μM, colony formation was less than that of the control. In the 5 and 10 μM groups, colony formation was less than that in the control group. There was a significant difference between the groups (* *p* < 0.05, ** *p* < 0.01). **TSC-3C** had excellent inhibitory activity against MDA-MB-231 colony formation.

### 2.4. Effect of TSC-3C on MDA-MB-231 Cell Migration

**Figure 3:**
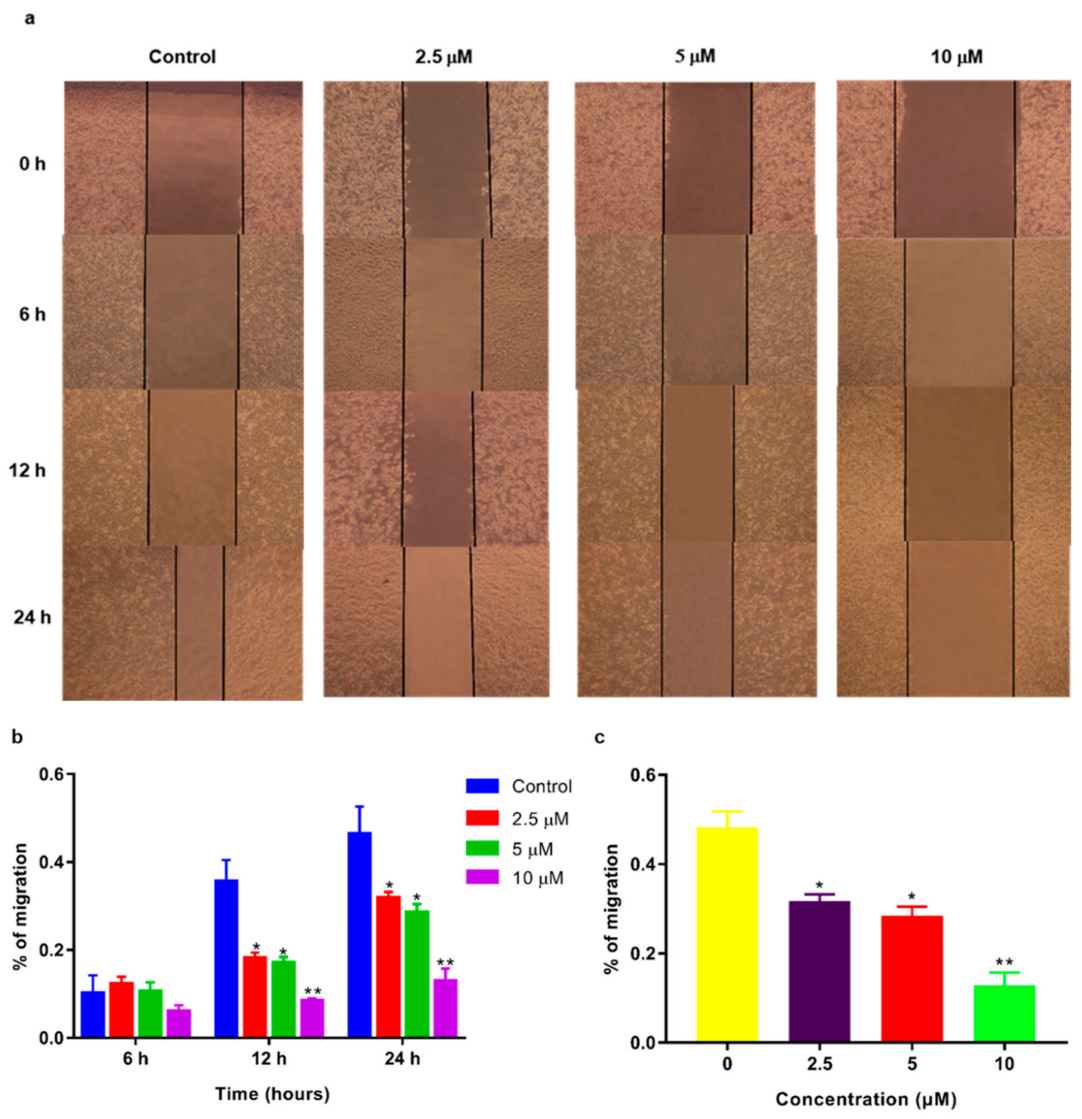
After treatment with **TSC-3C**, MDA-MB-231 cells were scratched and observed for a given time. (**a**) Images of the wounds over time in cells treated with different concentrations of **TSC-3C** (100×). (**b**) Histogram showing the migration rate at different times compared with that at 0 h. (**c**) Histogram of the MDA-MB-231 cell migration rate after 24 h. The data are expressed as the means ± SD (*n* = 3). * *p* < 0.05 vs. the control group, ** *p* < 0.01 vs. the control group.

After culturing cells with a concentration gradient of **TSC-3C**, we observed the wound scratch at the same site over time. As shown in Figure 3a, the control group showed a 50.6% ± 0.72 migration rate compared with the width of the wound scratch at 0 h. The 2.5 μM group displayed a 31.8% ± 1.03 migration rate after 24 h. The 5 μM group had a 28.4% ± 1.53 migration rate after culture for 24 h. In the group treated with a high concentration of 10 μM, only 13.4% ± 0.87 of cells migrated after 24 h. The migration rate at different concentrations is shown in Figure 3c. The migration of cells at 0, 6, 12, and 24 h is shown in Figure 3b. The MDA-MB-231 cell migration rate was increasingly inhibited by increasing concentrations of **TSC-3C**.

### 2.5. Inhibition of MDA-MB-231 Cell Invasion

**Figure 4:**
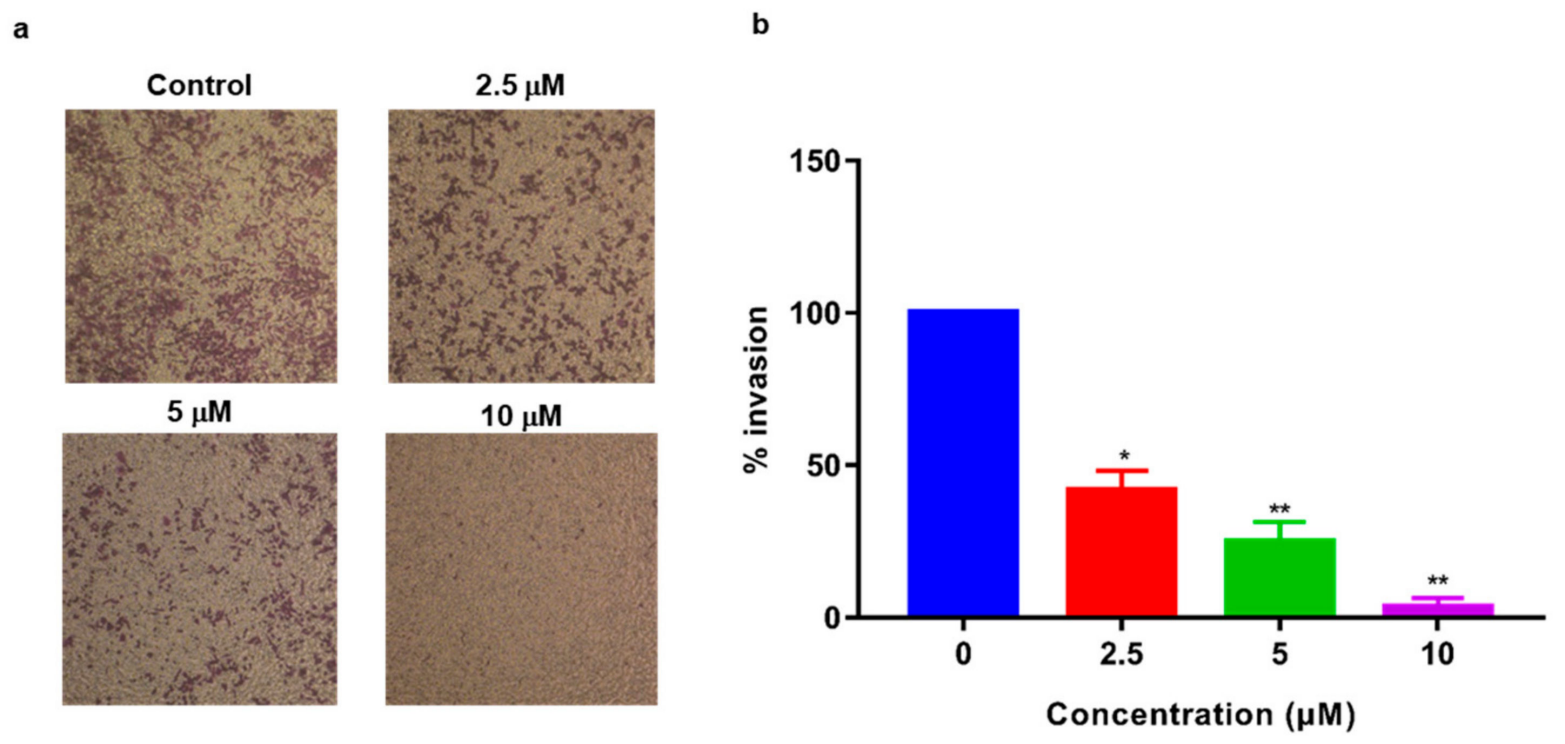
(**a**) MDA-MB-231 cell invasion was affected after culturing with **TSC-3C** at various concentrations (100×). (**b**) Histogram of the inhibition rate of **TSC-3C** in MDA-MB-231 cell invasion. The data are expressed as the means ± SD from three independent experiments. * *p* < 0.05 vs. the control group, ** *p* < 0.01 vs. the control group.

MDA-MB-231 cells were cultured in transwells coated with Matrigel, for 48 h, and treated with gradient concentrations of **TSC-3C**. The number of treated cells that invaded the transwell chamber were compared that of control cells. As shown in Figure 4, low **TSC-3C** concentration resulted in a 57% ± 1.54 inhibition rate after 48 h, while the 10 μM drug treatment completely inhibited the invasion of MDA-MB-231 cells.

### 2.6. TSC-3C Decreased the Mitochondrial Membrane Potential

After treatment with various concentrations of **TSC-3C**, we stained MDA-MB-231 cells with JC-1. JC-1 is an ideal fluorescent probe that is widely used in the detection of mitochondrial membrane potential. When the mitochondrial membrane potential is high, JC-1 aggregates in the mitochondrial matrix to form polymer/J-aggregates, which can produce red fluorescence. When the mitochondrial membrane potential is low, JC-1 cannot be concentrated in the mitochondrial matrix. In this case, JC-1 is a monomer and can produce green fluorescence. The relative ratio of red:green fluorescence is used to measure the ratio of mitochondrial depolarization.

**Figure 5:**
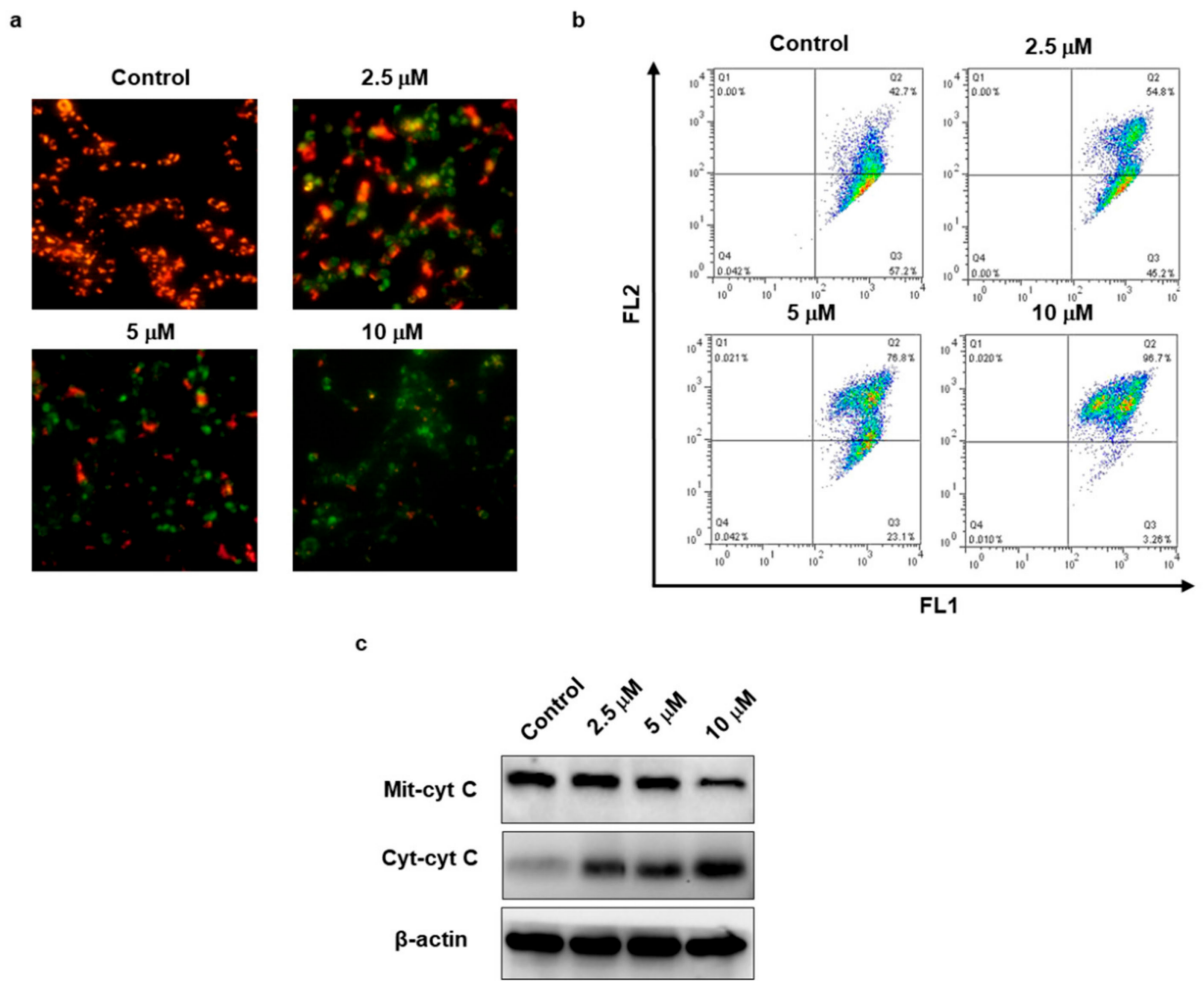
**TSC-3C** disrupts the mitochondrial membrane potential of MDA-MB-231 cells. (**a**) Mitochondrial membrane potential of MDA-MB-231 cells treated with various concentrations of **TSC-3C** and observed under fluorescence microscopy (200×). (**b**) MDA-MB-231 cells were treated with various concentrations of **TSC-3C** for 48 h. The cells were stained with JC-1 and analyzed by FACS. (**c**) MDA-MB-231 cells were cultured with **TSC-3C** for 48 h. Mitochondrial and cytoplasmic proteins were extracted. Western blotting was used to detect the level of cytochrome C. β-Actin was used to normalize the protein content.

Under fluorescence microscopy, the red color decreased with an increase in green fluorescence, indicating that the mitochondrial membrane potential was reduced in MDA-MB-231 cells. The results are shown in Figure 5a. **TSC-3C** reduced the mitochondrial membrane potential of MDA-MB-231 cells. In the FACS (flow cytometry) assay, compound **TSC-3C** at 2.5 μM decreased the mitochondrial membrane potential by 11.9%, as compared to the NC group. Treatment with 5 μM reduced the mitochondrial membrane potential of MDA-MB-231 cells by 34.1%. The 10 μM group had the highest decrease in mitochondrial membrane potential, which was 54% more than that of the control group, as shown in Figure 5b. After treatment with **TSC-3C** for 48 h, Western blot analysis showed that mitochondrial cytochrome C in MDA-MB-231 cells decreased, and cytochrome C in the cytoplasm increased in a dose-dependent manner, suggesting that the mitochondrial membrane was destroyed by **TSC-3C**.

### 2.7. TSC-3C Induces Apoptosis in MDA-MB-231 Cells

**Figure 6:**
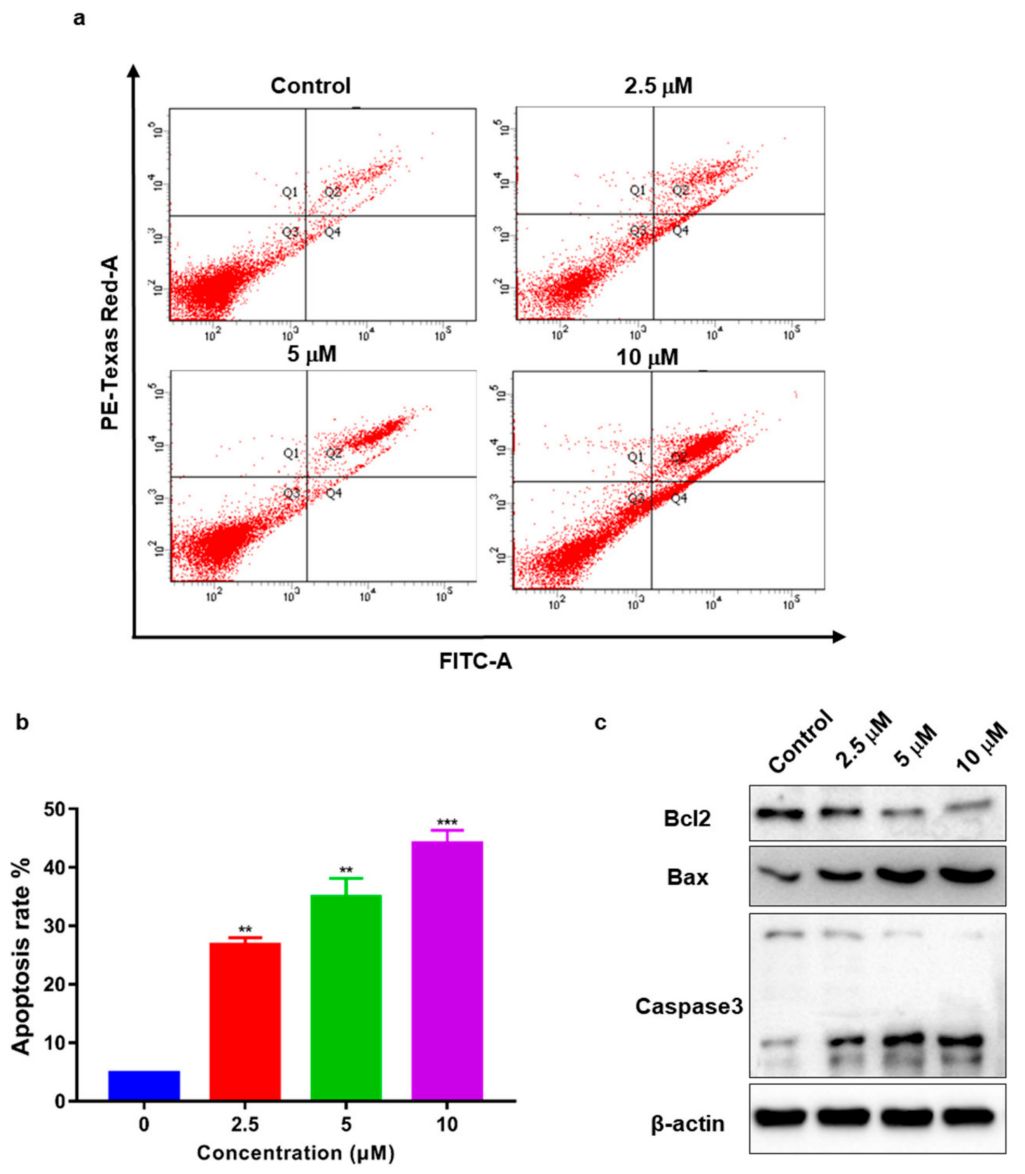
**TSC-3C** induces apoptosis in MDA-MB-231 cells. (**a**) After treatment with various concentrations of **TSC-3C** for 48 h, the cells were stained with Annexin V/PI and analyzed, using FACS. (**b**) After treatment with **TSC-3C** for 48 h, MDA-MB-231 cells were analyzed for the levels of Bcl2, Bax, and caspase-3 by Western blot. (**c**) Histogram showing the apoptotic rate of the experimental group compared with the control group. The data are expressed as the means ± SD from three independent experiments. ** *p* < 0.01 vs. the control group, *** *p* < 0.001 vs. the control group.

After treatment with various concentrations of **TSC-3C**, we stained MDA-MB-231 cells with JC-1. Under fluorescent electron microscopy, the red color decreased with an increase in green fluorescence. Furthermore, after treatment with various concentrations of **TSC-3C** for 48 h, the effects of **TSC-3C** on cell survival were investigated. The results showed that **TSC-3C** could induce apoptosis in MDA-MB-231 cells (Figure 6). The rate of apoptosis was increased with increasing concentrations of **TSC-3C**, and this is shown in Figure 6b. In addition, the expression of several classic markers of apoptosis, such as Bax, Bcl2, and caspase-3, was determined by Western blotting. The level of Bcl2 protein was decreased, while the level of Bax was increased in **TSC-3C**-treated MDA-MB-231 cells. In addition, caspase-3 was upregulated by **TSC-3C**, in MDA-MB-231 cells (Figure 6c).

### 2.8. TSC-3C Affects the MAPK Pathway

**Figure 7:**
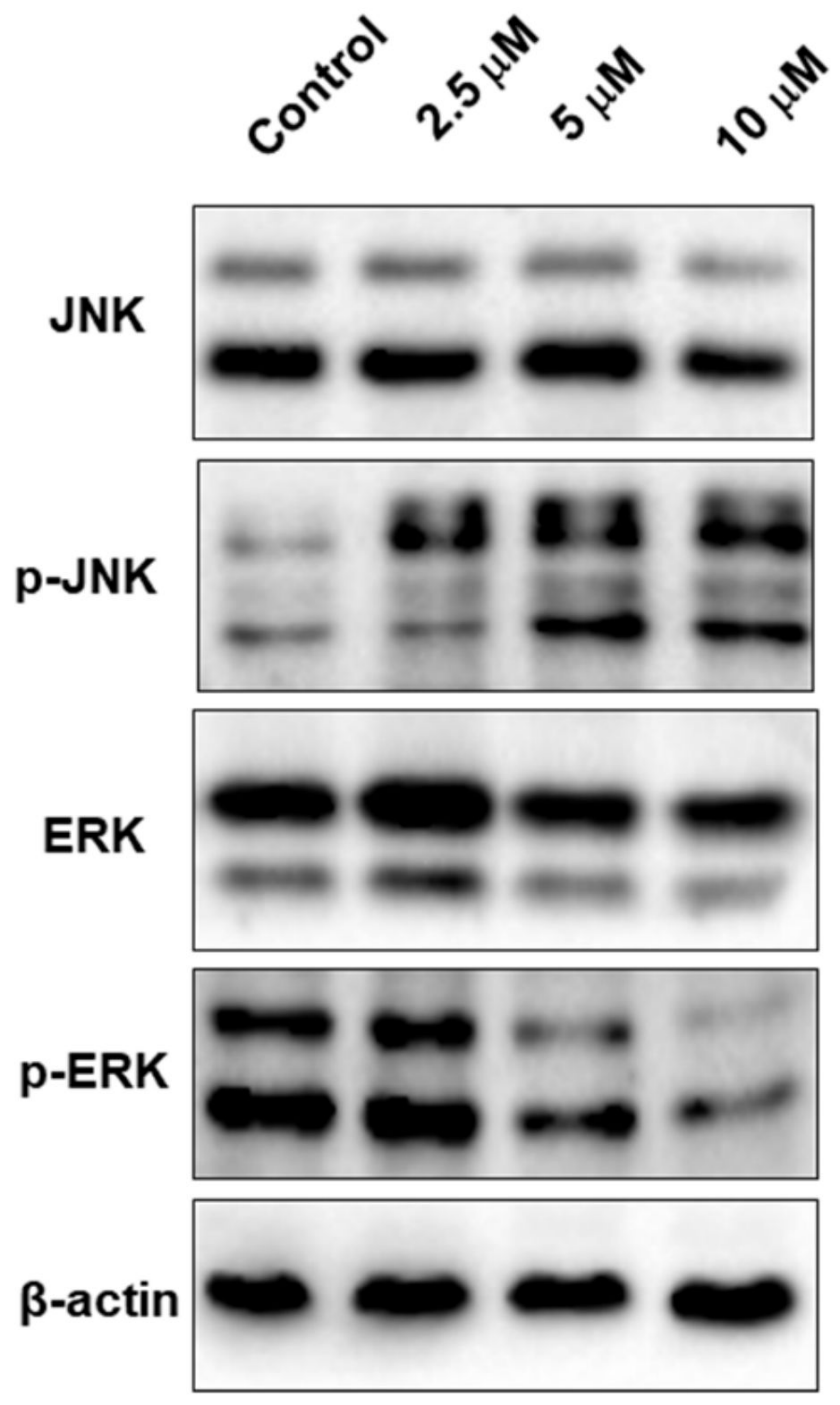
MDA-MB-231 cells were cultured with **TSC-3C** for 48 h, and the MAPK protein was analyzed by Western blot. β-Actin was used to normalize the protein content.

As mentioned previously, MAPK pathways are involved in many cancer processes. After MDA-MB-231 cells were treated with **TSC-3C**, the total protein levels of p44/42 MAPK (ERK1/2) and JNK were not affected. However, phosphorylated JNK, which induces cell apoptosis, was increased by **TSC-3C**. Phosphorylated p44/42 MAPK (ERK1/2) was suppressed by **TSC-3C** in a dose-dependent manner (Figure 7).

## 3. Discussion

TNBC is one of the most aggressive malignant neoplasms, characterized by the lack of receptor targets, which limits the therapeutic options because it does not respond to conventional hormonal interventions. Currently, molecular-based targeted therapies have been proven to be the most important strategies to treat many aggressive cancers [46].

As expected, compounds that incorporated fluorine atoms showed potent activities against the tested cancer cell lines, especially **TSC-3C** in MDA-MB-231 cells. **TSC-3C** significantly inhibited the proliferation of MDA-MB-231 cells to a greater extent than that of other cell lines (Table 1). The results on cell colony formation demonstrated the anticancer activity of **TSC-3C**. MDA-MB-231 cells, which have strong migration and invasion abilities, were treated with **TSC-3C**, and the migrated and invaded cells in the transwell and wound scratch assays were decreased in a dose-dependent manner, indicating that the migration and invasion abilities of MDA-MB-231 cells were inhibited by **TSC-3C.**

Mitochondria are the energy-producing factories of the cell. After treatment with **TSC-3C**, the mitochondrial membrane of MDA-MB-231 cells was destroyed, and the mitochondrial potential decreased. This is shown in Figure 4. Then, the levels of cytochrome C in the mitochondria and cytoplasm showed that mitochondrial function was inhibited. Mitochondrial dysfunction leads to cell apoptosis. In addition, the levels of Bcl2, Bax and caspase 3 were detected by Western blot (Figure 6c). This result was consistent with the apoptosis results measured by using flow cytometry, showing that **TSC-3C** induced MDA-MB-231 apoptosis (Figure 7) through the mitochondrial apoptosis pathway. These data indicated that **TSC-3C**-induced apoptosis was caspase-dependent. By Western blot assay, for MDA-MB-231 cells, we found changes in the levels of several proteins in the MAPK pathway. The level of p-JNK decreased and p-p44/42 MAPK (ERK1/2) increased after treatment with **TSC-3C**. The result will induce cell apoptosis [39,40,42]. In other words, **TSC-3C** inhibited MDA-MB-231 cell proliferation, migration, and invasion and induced a decrease in the mitochondrial membrane potential, which may result from changes in the MAPK pathway. In conclusion, our results suggest that **TSC-3C** could be a good candidate for targeted therapy for triple negative breast cancer (TNBC).

Overall, **TSC-3C,** which was modified by the incorporation of fluorine atoms, had potent activities in TNBC, evidenced by the excellent anticancer activity against MDA-MB-231 cells. Considering its biological effects, this compound appears to be worthy of further analysis, to demonstrate its efficacy as a novel anticancer agent.

## 4. Materials and Methods

### 4.1. Chemistry

#### 4.1.1. Procedure for the Synthesis of Compound **2**

A solution of thiosemicarbazide (4 g, 44 mmol, 1.1 equiv) in ethanol (95%, 45 mL) was refluxed with 3,4-difluorobenzaldehyde (5.6 g, 40 mmol, 1.0 equiv), in the presence of a few drops of glacial acetic acid. TLC (Thin-Layer Chromatography) analysis indicated when the reaction was complete. The precipitate that formed after cooling to room temperature was collected by filtration and washed with adequate amounts of ethanol and dried under a vacuum to give compound **2**.

#### 4.1.2. Procedure for the Synthesis of Compound **4**

A solution of 3,4-difluoroacetophenone (0.78 g, 5 mmol, 1.0 equiv) in EtOAc (20 mL) was refluxed with copper bromide (2.23 g, 10 mmol, 2.0 equiv) at 90 °C. TLC analysis indicated when the reaction was complete. The filtrate filtered by diatomite was washed with water and dried over Na_2_SO_4_. The solvent was removed under vacuum, to give an oil, and the product was used directly for the next step, without further purification.

#### 4.1.3. Procedure for the Synthesis of Compound **5**

A mixture of 3,4-difluorothiosemicarbazone (**2**, 1.05 g, 5 mmol, 1.0 equiv), anhydrous sodium acetate (1.64 g, 20 mmol, 4.0 equiv), and 20 mL ethanol was stirred in a 100 mL round-bottom flask for 10 min. Then, 3,4-difluoroacetophenone (**4**, all obtained in step b) was added, and the reaction was refluxed at 90 °C. TLC analysis indicated when the reaction was complete. After cooling back to r.t., the precipitate was collected by filtration, washed with adequate amounts of ethanol, and dried under vacuum, to give compound **5**. The crude product was purified by recrystallization in ethanol.

### 4.2. Cell Lines and Cell Culture

The cell lines used were all purchased from the Chinese Academy of Science (Shanghai, P.R. China). The human breast cancer cell line MDA-MB-231, human ovarian cancer cell line SK-OV-3, A549 (human lung cancer cell line)s, and PANC-1 (human pancreatic cell line) were cultured with Dulbecco’s modified Eagle’s medium supplemented with 10% fetal bovine serum (FBS), 100 U/mL penicillin, and 100 mg/mL streptomycin. The HepG2 (human hepatoma cell line) and U87 MG (human primary glioblastoma cell line) were cultured in Eagle’s minimum essential medium (MEM) supplemented with 10% FBS, 100 U/mL penicillin, and 100 U/mL streptomycin. The human liver cell line L-02 was cultured in Roswell Park Memorial Institute-1640 supplemented with 10% FBS. The human colonic epithelial cell line Caco-2 was cultured in minimum essential medium with 10% FBS. The human breast cancer cell line MCF-7 and human pancreatic cell line Capanc-1 were cultured with Dulbecco’s modified Eagle’s medium supplemented with 20% fetal bovine serum (FBS). All cells were cultured at 37 °C in humidified CO_2_ (5%).

### 4.3. Cell Proliferation Assay

Cells (MDA-MB-231, A549, HepG2, U87 MG, PANC-1, MCF-7, SK-OV-3, Caco-2, Capanc-1, and L-02) were seeded in 96-well plates at a density of 3000 cells per well and allowed to settle for 24 h. Then, the cells were treated with varying concentrations of **TSC-3C** (0, 2.5, 5, 10 μM). After 48 h, add M TT (5 mg/mL) to the plates and incubated at 37 °C for 4 h. Supernatant was removed, and the formed crystals were dissolved in 150 μL DMSO and measured by a microplate reader (BioTek, Winooski, VT, USA) at 490 nm. Experiments were performed in triplicate, with six wells for each measurement.

### 4.4. Colony Forming Assay

Seed MDA-MB-231 cells in 6-well plates at a density of 500 cells per well. After 24 h, cells were treated with **TSC-3C** (0, 2.5, 5, 10 μM), and the cells were incubated for 11 days, to allow proliferate. After staining with crystal violet, colonies containing more than 50 cells were counted and evaluated.

### 4.5. Cell Migration Assay

First, MDA-MB-231 cells were seeded in 12-well plates, at a density of 3 × 10^5^ cells per well, and allowed to settle overnight. Then, the cells attached to the bottom of the well were scratched to make a wound scratch perpendicular to the marker line. Next, the floating cells were washed off. DMEM was supplemented with various concentrations (0, 2.5, 5, 10 µM) of **TSC-3C**. The wound scratch was observed exactly at 0, 6, 12, and 24 h. The wound width was measured at different times, and the inhibitory effect of **TSC-3C** on MDA-MB-231 cell migration was analyzed.

### 4.6. Cell Invasion Assay

Each chamber of the Transwell plate was coated with 20 mL of Matrigel diluted in DMEM (1:7) and allowed to harden. Then, MDA-MB-231 cells were seeded at a density of 5 × 10^5^/100 μL overnight. DMEM in the chamber was supplemented with **TSC-3C** (0, 2.5, 5, 10 μM). The bottom well of the plate was filled with 500 μL DMEM with 10% FBS. After 48 h, the cells on the top of the chamber were removed, the cells on the other side of the chamber were washed, and the cells were fixed with paraformaldehyde. After drying with air, the cells were stained with crystal violet. Images were obtained to analyze the invasive cells on the back of chamber.

### 4.7. Measurement of Mitochondrial Membrane Potential (Δφm)

For mitochondrial membrane potential (Δφm) analysis, MDA-MB-231 cells were treated with various concentrations (0, 2.5, 5, 10 μM) of **TSC-3C** for 48 h. The cells were stained with the fluorescent indicator JC-1 at 37 °C, for 15 min (mitochondrial membrane potential detection kit (JC-1, Cat. No. C2006, Beyotime, Beijing, China), and after three washes with PBS, the cells were harvested and analyzed, using fluorescence microscopy (Nikon, Shanghai, Japan)), and flow cytometry.

### 4.8. Fluorescence Probe JC-1 and Fluorescence Microscopy

JC-1 is a member of a family of lipophilic cations that translocate into mitochondria in response to potential. The cells harvested in Section 4.7 were analyzed by fluorescence microscopy at 488 and 590 nm. Upon excitation at 488 nm (FITC-related signals), the dye fluoresces green. The unique feature of this probe is that, at high concentrations, it forms so-called J complexes, whose emission spectrum shifts to red, with a peak at 590 nm. Thus, theoretically, it can be used as a ratiometric dye to measure the ratio between red and green fluorescence as an indicator of mitochondrial membrane potential. We acquired images of the different groups of cells harvested in Section 4.7 by using a fluorescence microscopy camera (Nikon, Shanghai, Japan).

### 4.9. Detection of Apoptosis

MDA-MB-231 cells were treated with different concentrations of **TSC-3C** for 48 h. The cells were harvested. In the dark, they were stained with Annexin V/PI for 15 min. Then the cells were analyzed by flow cytometry.

### 4.10. Western Blotting

After treatment, whole-cell lysates of MDA-MB-231 cells were extracted with RIPA buffer supplemented with EDTA free Protease Inhibitor Cocktail (Roche, Shanghai, China) and PhosSTOP Phosphatase Inhibitor (Roche, Shanghai, China). We used a bicinchoninic acid (BCA) protein assay kit (Thermo Fisher scientific, Shanghai, China) to quantify the protein contents of the cell lysates and Bradford protein assay to measure protein concentrations. Equal amounts of protein samples were separated by SDS-PAGE and transferred to PVDF membranes, followed by immunoblotting with primary antibodies against cytochrome C (Cell Signaling Technology, Cat. No. 11940S, Danvers, MA, USA), caspase 3 (Cell Signaling Technology, Cat. No. 14220S, Danvers, MA, USA), JNK (Cell Signaling Technology, Cat. No. 9252, Danvers, MA, USA), p-JNK (Santa Cruz Biotechnology Cat. No. L2718, Danvers, MA, USA), p44/42 MAPK (ERK1/2) (Cell Signaling Technology, Cat. No. 4696S, Danvers, MA, USA), p-p44/42 MAPK (ERK1/2) (Cell Signaling Technology, Cat. No. 4370T, Danvers, MA, USA), Bcl2 (Cell Signaling Technology Cat. No. 4223S, Danvers, MA, USA), Bax (Cell Signaling Technology Cat. No. 5023S, Danvers, MA, USA), β-actin (Proteintech, Cat. No. 60008-I-Ig, Rosemont, PA, USA), and GAPDH (Proteintech Cat. No. 60004-I-Ig, Rosemont, PA, USA), overnight, at 4 °C. The next day, the membranes were washed with TBST three times for 5 min. The cells were incubated with horseradish peroxidase (HRP)-conjugated secondary antibodies. The bands were detected by using the BeyoECL enhanced chemiluminescence system (Beyotime, Beijing, China). The Versa DOC imaging system (BioRad, Hercules, CA, USA) was used to visualize the protein signals.

Immunoblots were quantified by densitometry, and the data were expressed as arbitrary units (protein/β-actin).

### 4.11. Statistical Analysis

Statistical analysis was performed using GraphPad Prism 7.0 (San Diego, CA, USA). Differences were considered statistical significantly at p < 0.05. P < 0.01 was considered statistically significant extremely. The data were presented as mean ± SD.

## Figures and Tables

**Table 1:** IC_50_ of **TSC-3C** in a variety of cell lines. The data are expressed as the means.

Cell Lines	IC_50_ (μM)
MDA-MB-231	2.79 ± 0.71
A549	8.12 ± 1.22
Capanc-1	8.45 ± 1.21
Caco-2	12.33 ± 1.31
MCF-7	22.77 ± 1.16
PANC-1	26.32 ± 1.35
HepG2	27.10 ± 1.98
SK-OV-3	33.81 ± 1.87
U87 MG	40.00 ± 2.03
L-02	not reached

## Abbreviations

TNBC	Triple negative breast cancer
ER	Estrogen receptor
PR	Progesterone receptor
FBS	Fetal bovine serum
